# Increased resected lymph node stations improved survival of esophageal squamous cell carcinoma

**DOI:** 10.1186/s12885-024-11886-7

**Published:** 2024-02-05

**Authors:** Run-Da Lu, Zheng-Dao Wei, Yi-Xin Liu, Dong Tian, Han-Lu Zhang, Qi-Xin Shang, Wei-Peng Hu, Lin Yang, Yu-Shang Yang, Long-Qi Chen

**Affiliations:** Department of Thoracic Surgery, West China Hospital of Sichuan University, No. 37 Guoxue Alley, 610041 Chengdu, China

**Keywords:** Neoadjuvant chemoradiotherapy, Lymph node dissection strategy, Esophageal squamous cell carcinoma, Lymph node station, Survival

## Abstract

**Background:**

Neoadjuvant chemoradiotherapy (nCRT) and surgery have been recommended as the standard treatments for locally advanced esophageal squamous cell carcinoma (ESCC). In addition, nodal metastases decreased in frequency and changed in distribution after neoadjuvant therapy. This study aimed to examine the optimal strategy for lymph node dissection (LND) in patients with ESCC who underwent nCRT.

**Methods:**

The hazard ratios (HRs) for overall survival (OS) and disease-free survival (DFS) were calculated using the Cox proportional hazard model. To determine the minimal number of LNDs (n-LNS) or least station of LNDs (e-LNS), the Chow test was used.

**Results:**

In total, 333 patients were included. The estimated cut-off values for e-LNS and n-LNS were 9 and 15, respectively. A higher number of e-LNS was significantly associated with improved OS (HR: 0.90; 95% CI 0.84–0.97, *P* = 0.0075) and DFS (HR: 0.012; 95% CI: 0.84–0.98, *P* = 0.0074). The e-LNS was a significant prognostic factor in multivariate analyses. The local recurrence rate of 23.1% in high e-LNS is much lower than the results of low e-LNS (13.3%). Comparable morbidity was found in both the e-LNS and n-LND subgroups.

**Conclusion:**

This cohort study revealed an association between the extent of LND and overall survival, suggesting the therapeutic value of extended lymphadenectomy during esophagectomy. Therefore, more lymph node stations being sampled leads to higher survival rates among patients who receive nCRT, and standard lymphadenectomy of at least 9 stations is strongly recommended.

**Supplementary Information:**

The online version contains supplementary material available at 10.1186/s12885-024-11886-7.

## Backgroud

Esophageal squamous cell carcinoma (ESCC) is the most common type of EC in Asia [1]. Radical esophagectomy with lymphadenectomy is the principal treatment for ESCC [2]. Moreover, many studies have shown that neoadjuvant therapy before surgical resection improves the long-term survival of patients with resectable locally advanced ESCC [3–5]. While neoadjuvant chemoradiotherapy (nCRT) with surgery has gradually become the standard treatment, recurrence after nCRT remains high, with recurrence rates of > 40% [4, 6–8]. Therefore, it is crucial to further optimize the treatment for patients with locally advanced ESCC.

Multiple studies proposed that lymph node metastasis (LNM) is the dominant prognostic factor of ESCC [9]. The AJCC cancer staging system utilize the number of metastatic lymph nodes (LNs) as the criterion for node staging. Furthermore, to achieve more accurate N staging, this guideline recommends that adequate lymphadenectomy should include resection of 12–22 nodes [10]. It is noteworthy that the 8th AJCC Cancer Staging System was developed mainly for patients without preoperative therapy [11]. Additionally, numerous studies have demonstrated a decrease in nodal metastasis frequency and alterations in nodal metastasis distribution after neoadjuvant therapy [12–14]. In clinical practice, the extent of lymph node dissection for esophageal squamous cell carcinoma (ESCC) varies depending on the surgeon’s experience, potentially leading to variations in the dissection scope. Pathologists may encounter difficulties in accurately assessing lymph nodes, such as the fusion of multiple positive nodes into a single macroscopic mass or the fragmentation of one enlarged node into several pieces during surgical resection [15, 16]. Staging ESCC patients solely based on the number of excised lymph nodes cannot fully capture the complexity of the disease. A readily available and reliable system is needed. Additionally, we know that nCRT not only reduces the number of affected lymph nodes but also modifies the frequency of node site involvement, it is unclear whether the number of lymph node dissections or the number of dissection stations is a more effective predictor [5, 17–22]. This study aims to explore the optimal number of LNDs (n-LNDs) and the station of LNDs (e-LNSs) in patients with ESCC undergoing nCRT followed by esophagectomy.

## Methods

### Study design and patients

This single-center retrospective cohort study was conducted to explore the relationship between e-LNS/n-LND and the survival of patients with ESCC who underwent nCRT. We reviewed the records of 396 patients from the esophageal carcinoma database of the West China Hospital between February 2016 and March 2021.The inclusion criteria were as follows: (1) pathologically confirmed thoracic ESCC; (2) neoadjuvant therapy; (3) age 18–80 years; (4) pathological stage ypT0N0MO-ypT4bN3M0 (stages I–IVA) according to the eighth edition of the AJCC Cancer Staging Manual; (5) no distant metastasis (M0); (6) tumor-free resection margins (R0); and (7) complete follow-up data. Patients with a history of other malignant tumors or death within 30 days of surgery were excluded.

This study was approved by the Ethics Committee of West China Hospital of Sichuan University (2,019,632), which waived the requirement for written informed consent from individual patients because of the retrospective nature of this study.

### Treatment procedure

Neoadjuvant chemotherapy consisted of paclitaxel plus cisplatin or 5-fluorouracil plus cisplatin, along with concomitant radiotherapy at a dosage of 45 Gy. Surgical feasibility after 2–4 cycles of neoadjuvant treatment was determined through preoperative assessment. Esophagectomy was performed using the McKeown or Ivor Lewis procedure, depending on the location and extent of the tumor. In this study, cervical LND was highly selected for patients with suspected cervical LN metastasis according to computed tomography and ultrasonography taken prior to surgery. We routinely conducted two-field LND (thoracic and abdomen) in patients without preoperative detection of cervical lymph node metastases.

### Surgery quality control

To ensure quality control of the operation, all surgeries were performed in high volume centers with a long history of performing esophagectomies (≥ 200 esophagectomies/year). The esophagectomies were required to be performed by experienced senior surgeons in their preferred approach. Surgical quality control is evaluated by using intraoperative video recordings and the resection margin of the specimens.

### Study outcomes and follow-Up

The primary outcome was overall survival (OS), which was calculated from the date of surgery to the date of death, and disease-free survival (DFS), which was measured until the occurrence of the first recurrence or death from any cause. The secondary outcomes were recurrence, metastasis rates and morbidity.

All patients were telephonically followed up every 3 months for the first 2 years, every 6 months for the next 3 years, and annually thereafter. Patients who were still alive or lost to follow-up were censored at the date of their last follow-up. The last general follow-up of survivors was performed in September 2022. Follow-up information was available 3 years after surgery or at the time of death.

### Statistical analysis

Normal continuous variables are presented as the mean ± standard deviation, and nonnormal continuous variables are presented as the number of observations (N) or median and interquartile range (IQR) (25–75% percentile). For the descriptive statistical variables, we calculated 95% confidence intervals (CIs). The t test or nonparametric test (e.g., Wilcoxon test) was used to compare data between two groups. The number of patients and frequency (percentage) of each kind of discontinuous variable were calculated utilizing descriptive statistics. Pearson’s chi-square test, the Wilcoxon signed-rank test, and Fisher’s exact test were used to compare data between two groups. The Kaplan–Meier method was used to estimate OS and DFS, and the log-rank test was used to compare them between the groups.

Univariate and multivariate analyses with a Cox proportional hazard model were performed to assess the effect of different factors on OS and DFS, and these data are expressed as hazard ratios (HRs). The association of the examined lymph node station (e-LNS) (or examined lymph node [ELN]) count with OS was investigated using a multivariable Cox proportional hazards regression model. All clinicopathological and statistically significant factors were included for adjustment in both multivariable models to avoid confounding factors. The HR generated by the multivariable model was fitted using locally weighted scatterplot smoothing with a default bandwidth of 2/3 to visualize the correlations of higher e-LNS (ELN) counts with OS and DFS. The Chow test was used to determine the structural breakpoints of e-LNS (ELN), as previously described by Liang et al [23]. In our study, the cut-off points of e-LNSs and E-LNSs were recognized, taking into account the clinical effect of the threshold for optimal survival.

SPSS, version 26.0 (IBM Corp., Armonk, NY) and R statistical language, version 4.1.2 (The R Project for Statistical Computing), were used to perform statistical analyses. All tests were set at a 2-sided *P* value < 0.05.

## Results

### Patient characteristics

**Fig. 1:**
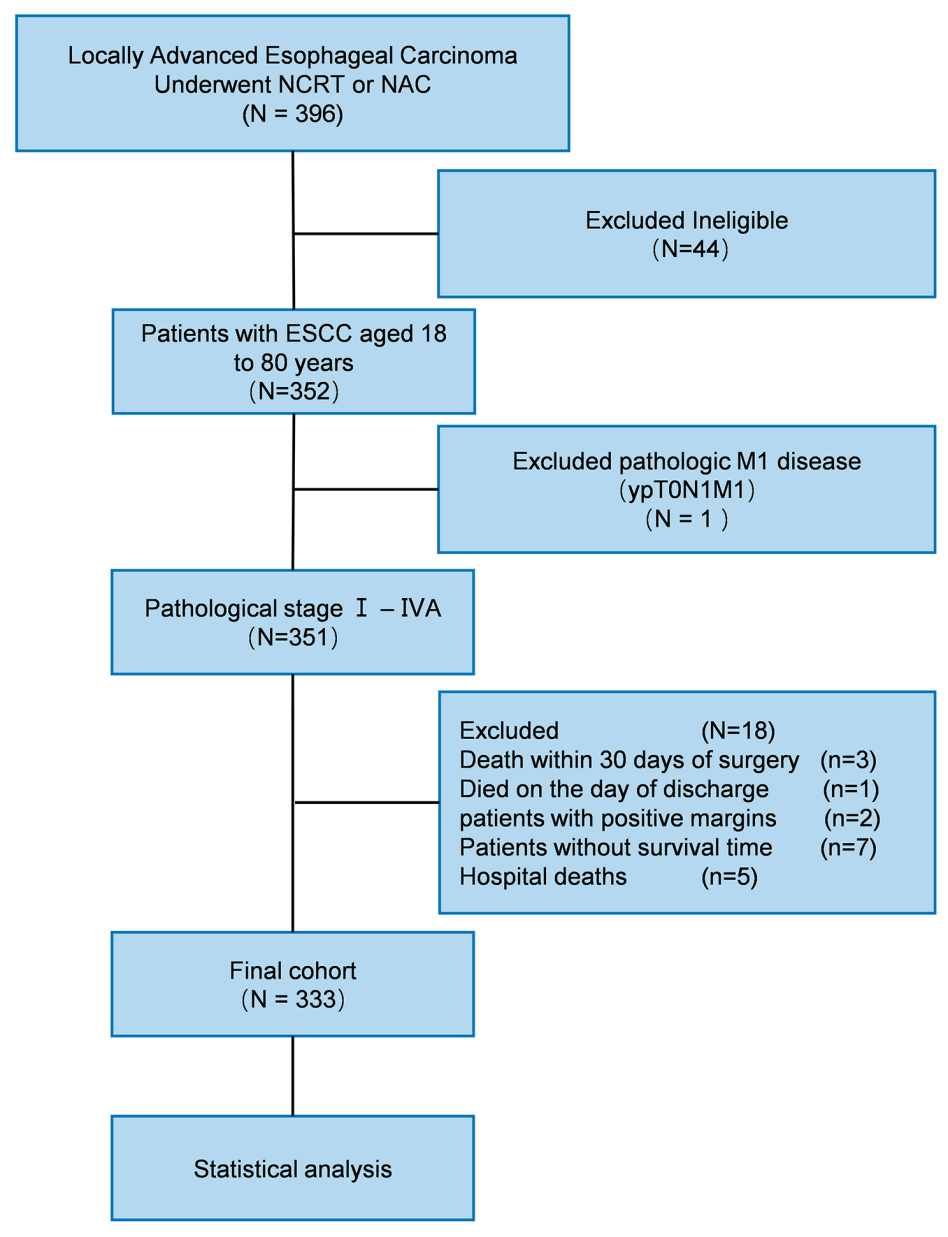
Flow chart of patient selection. ESCC, esophageal squamous cell carcinoma; nCRT, neoadjuvant chemoradiotherapy; NAC, neoadjuvant chemotherapy

The current study included 333 patients who underwent esophagectomy with neoadjuvant treatment (Fig. 1). The patients’ overall median age was 63 years (IQR, 56–68 years), including 266 (79.9%) men and 67 (20.1%) women. Upon histologic examination, 211 patients (63.4%) had no metastatic LNs (ypN0), and 131 patients (39.3%) had no live tumor cells in the esophageal wall (ypT0). The pathological complete response (TRG 0) rate was 39.3%, as shown in Table 1. The OS rates at 1 and 3 years were 84.3 and 62.5%, respectively.

**Table 1:** Baseline Demographic Characteristics of ESCC Patients who Underwent NCRT

Characteristics	Participants, No. (%)						
	Total Cohort (*N* = 333)	Number of e-LNS		*P* Value	Number of ELN		*P* Value
		≤ 9 (*n* = 108)	> 9 (*n* = 225)		≤ 15 (*n* = 86)	> 15 (*n* = 247)	
Age, years, median [IQR]	63[56–68]	62[55–68]	63.0[56–68]	0.502*	63[55–68]	63[56-67.5]	0.754*
≤ 60	126(37.8)	42(38.9)	84(37.3)	0.878	33(38.4)	93(37.7)	1
>60	207(62.2)	66(61.1)	141(61.1)		53(61.6)	154(62.3)	
Sex							
Female	67(20.1)	21(19.4)	46(20.4)	0.947	20 (23.3)	47 (19.0)	0.493
Male	266(79.9)	87(80.6)	179(79.6)		66 (76.7)	200 (81.0)	
Tumor location							
Proximal third	42(12.6)	8 (7.4)	34 (15.1)	0.099	5 (5.8)	37 (15.0)	0.085
Middle third	162(48.6)	59 (54.6)	103 (45.8)		46 (53.5)	116 (47.0)	
Distal third	129(38.7)	41 (38.0)	88 (39.1)		35 (40.7)	94 (38.1)	
Tumor length, cm median [IQR]	2.00 [0-3.6]	2.85 [0–4.0]	2.00 [0-3.5]	0.114*	2.50 [0-3.9]	2.00 [0-3.6]	0.716*
Differentiation							
Gx	138 (41.4)	34 (31.5)	104 (46.2)	0.033	34 (39.5)	104 (42.1)	0.374
G1	17 (5.1)	8 (7.4)	9 (4.0)		7 (8.1)	10 (4.0)	
G2	84 (25.2)	35 (32.4)	49 (21.8)		24 (27.9)	60 (24.3)	
G3	94 (28.2)	31 (28.7)	63 (28.0)		21 (24.4)	73 (29.6)	
pT							
0	131(39.3)	34(31.5)	97(43.1)	0.022	33 (38.4)	98 (39.7)	0.712
1	43 (12.9)	14 (13.0)	29 (12.9)		13 (15.1)	30 (12.1)	
2	45 (13.5)	11 (10.2)	34 (15.1)		9 (10.5)	36 (14.6)	
3	114 (34.2)	49 (45.4)	65 (28.9)		31 (36.0)	83 (33.6)	
pN							
0	211 (63.4)	70 (64.8)	141 (62.7)	0.647	57 (66.3)	154 (62.3)	0.809
1	80 (24.0)	26 (24.1)	54 (24.0)		19 (22.1)	61 (24.7)	
2	33 (9.9)	8 (7.4)	25 (11.1)		7 (8.1)	26 (10.5)	
3	9 (2.7)	4 (3.7)	5 (2.2)		3 (3.5)	6 (2.4)	
Pathological Stage							
1	163 (48.9)	48 (44.4)	115 (51.1)	0.18**	46 (53.5)	117 (47.4)	0.812**
2	48 (14.4)	22 (20.4)	26 (11.6)		11 (12.8)	37 (15.0)	
3	114 (34.2)	35 (32.4)	79 (35.1)		27 (31.4)	87 (35.2)	
4	8 (2.4)	3 (2.8)	5 (2.2)		2 (2.3)	6 (2.4)	
TRG							
0	131 (39.3)	34 (31.5)	97 (43.1)	0.16	33 (38.4)	98 (39.7)	0.769
1	40 (12.0)	12 (11.1)	28 (12.4)		13 (15.1)	27 (10.9)	
2	124 (37.2)	47 (43.5)	77 (34.2)		30 (34.9)	94 (38.1)	
3	38 (11.4)	15 (13.9)	23 (10.2)		10 (11.6)	28 (11.3)	
POLS, days, median (IQR)	11.0 [9-13]	11.0 [9-12]	11.0 [9-13]	0.889*	10.0 [9-12]	11.0 [9-13]	0.446*
Comorbidity							
NO	231 (69.4)	79 (73.1)	152 (67.6)	0.363	54(62.8)	177(71.7)	0.161
YES	102 (30.6)	29 (26.9)	73 (32.4)		32 (37.2)	70 (28.3)	

Abbreviation: ESCC, esophageal squamous cell carcinoma. NCRT, neoadjuvant chemoradiotherapy. E-LNS, examined lymph node station

ELN, examined lymph node. *, nonnormal. **, exact

### Cut-off point analysis

**Fig. 2:**
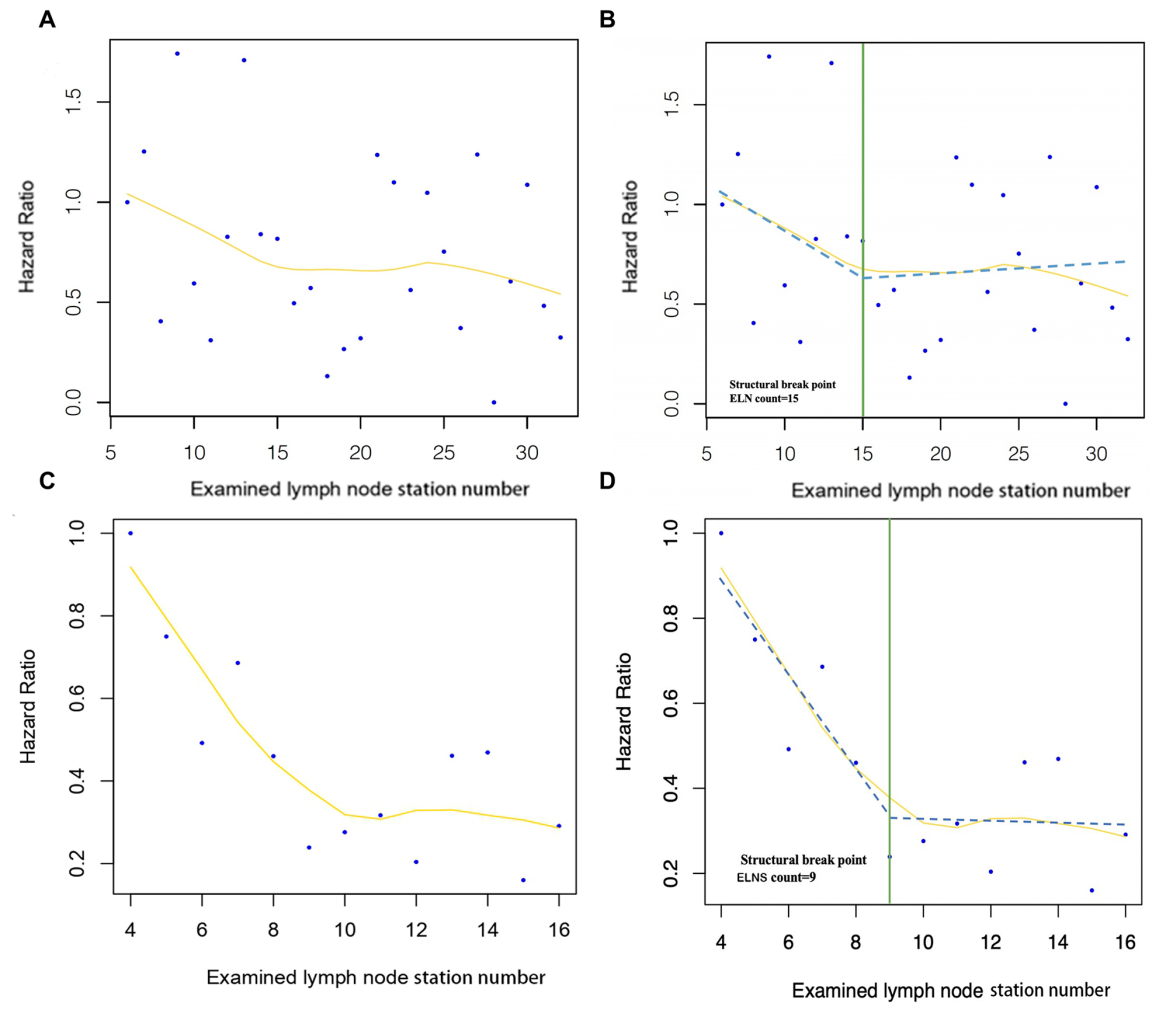
Association of resected lymph nodes with hazard ratios for OS. Overall survival fitted by LOWESS stratified by ELN (**A**). The structural break point determined by the Chow test stratified by ELN (**B**). Overall survival fitted by LOWESS stratified by E-LNS (**C**). The structural break point determined by the Chow test stratified by E-LNS (**D**). ELN, examined lymph node. E-LNS, examined lymph node station

To investigate the optimal number of lymph node dissections (n-LNDs), we used the Chow test to determine the structural cut-off to stratify patients into a high n-LND group or a low n-LND group. Meanwhile, the optimal extent of LND (e-LNS) was also explored based on the examined lymph node station, and patients were grouped as either high e-LNS or low e-LNS. According to the cut-off point analysis, the structural breakpoint of n-LND for the HR of OS was 15 (Fig. 2A, B), and e-LNS for the HR of OS was 9 (Fig. 2 C, D). Under the fitting curve of the HR mentioned above values, 9 was validated as the minimum threshold of e-LNS for the accurate prediction of optimal survival, and 15 was confirmed as the minimum threshold of n-LND.

### Effect of different LND strategies on OS

**Fig. 3:**
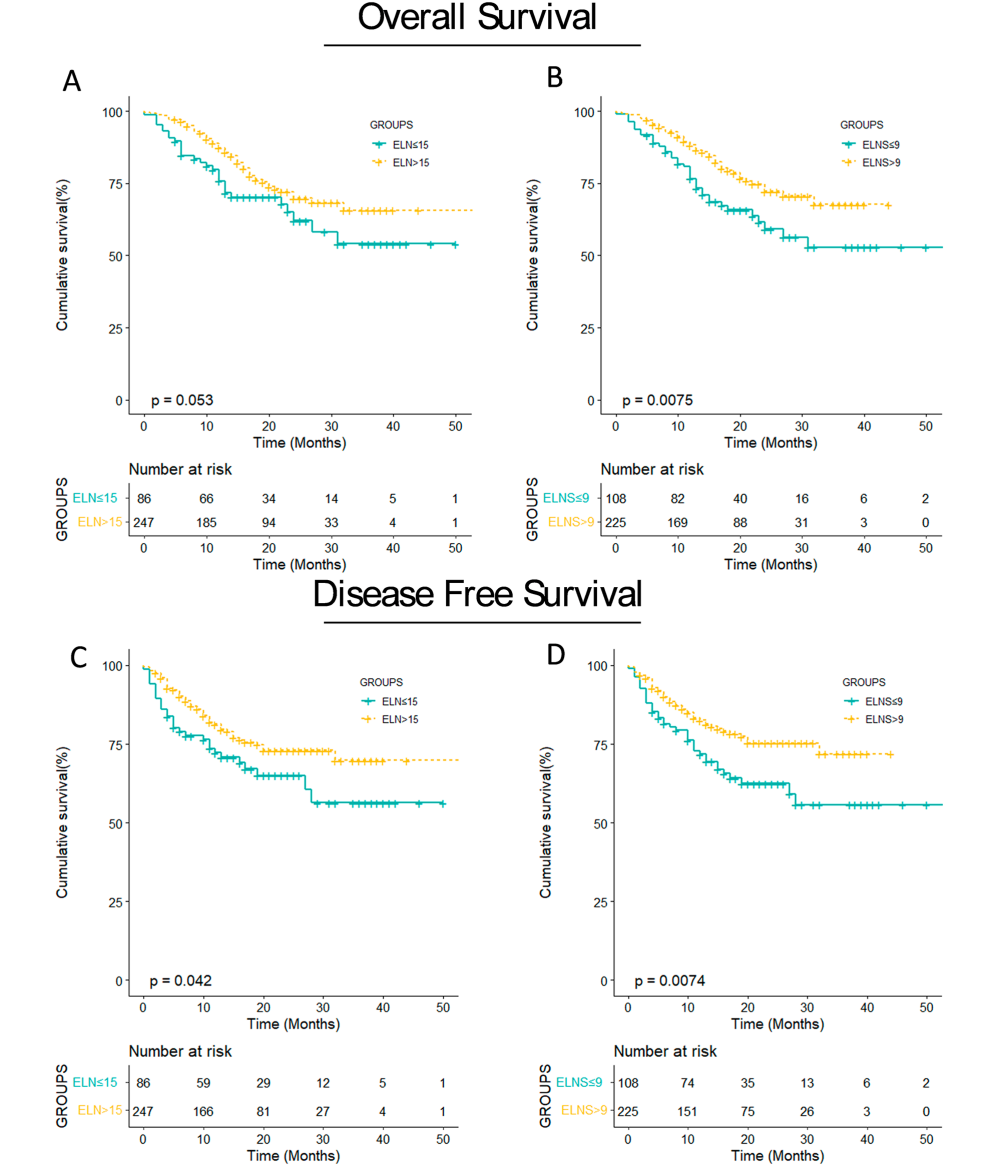
Kaplan‒Meier survival curve for OS and DFS. (**A**) Kaplan‒Meier survival curve for OS stratified by ELN after neoadjuvant therapy. (**B**) Kaplan‒Meier survival curve for OS stratified by E-LNS after neoadjuvant therapy. (**C**) Kaplan‒Meier survival curve for DFS stratified by ELN after neoadjuvant therapy. (**D**) Kaplan‒Meier survival curve for DFS stratified by E-LNS after neoadjuvant therapy. DFS, disease-free survival. OS, overall survival. ELN, examined lymph node. E-LNS, examined lymph node station

A comparison of the low e-LNS and high e-LNS groups is shown in Fig. 3. For OS, the survival curve of patients according to the e-LNS was significantly different between the groups (*P* = 0.0075), and the OS rates at 1 and 3 years were 76.6 and 52.8 for low e-LNS and 88.3 and 67.7 for high e-LNS. However, the survival analysis for n-LND observed no significant differences in OS between the two groups (*P* = 0.053) (Fig. 3A, B), and the OS rates at 1 and 3 years were 75.8% and 71.7% for low n-LND and 87.3% and 65.7% for high n-LND.

We used the Cox proportional hazard model to examine the independent impact between LND and survival. Univariate analysis was conducted for age (*P* = 0.169); sex (*P* = 0.158); differentiation (*P* = 0.000); n-LND (*P* = 0.428); e-LNS (*P* = 0.038); tumor location (*P* = 0.653); tumor length (*P* = 0.000); and ypT, ypN, and pathological stages (Table 2). Multivariate Cox regression analysis variation included differentiation, ypT, ypN, tumor length, e-LNS, and smoking. The e-LNS (HR: 0.90; 95% CI, 0.84–0.97; *P* = 0.007) was an independent factor affecting prognosis, as shown in Table 2.

**Table 2:** Univariate and multivariate Cox regression analysis of prognosis for OS in nCRT patients

Prognostic Factor	Univariable Analysis		Multivariable Analysis	
	HR (95% CI)	*P* Value	HR (95% CI)	*P* Value
Age, years	0.97(0.95-1)	0.169		
Sex	1.55(0.84–2.87)	0.158		
Differentiation				
Gx	1		1	
G1	1.27(0.37–4.32)	0.703	0.39(0.06–2.54)	0.323
G2	2.29(1.25–4.19)	0.007	0.56(0.12–2.70)	0.472
G3	3.60(2.05–6.32)	0	0.78(0.16–3.78)	0.759
ypT				
0	1		1	
1	1.59(0.65–3.87)	0.306	1.18(0.24–5.71)	0.840
2	1.86(0.86–3.99)	0.116	1.24(0.20–7.53)	0.816
3	3.83(2.18–6.73)	0	1.85(0.31–11.04)	0.498
ypN				
0	1		1	
1	3.34(2.02–5.52)	0	2.97(1.73–5.09)	**0**
2	3.50(1.85–6.60)	0	2.85(1.39–5.82)	**0.004**
3	18.38(8.13–41.60)	0	10.68(4.32–26.39)	**0**
Pathological Stage				
0	1			
1	2.51(25.58 − 0.4)	0.013		
2	4.7(25.58 − 0.4)	0		
3	25.58(25.58 − 0.4)	0		
Tumor location				
Proximal third	1			
Middle third	1.05(0.51–2.15)	0.897		
Distal third	1.11(0.53–2.34)	0.785		
Tumor length, cm	1.26(1.16–1.37)	0	1.13(0.98–1.31)	0. 099
ELN	0.99(0.97–1.02)	0.428		0.84
E-LNS	0.93(0.871)	0.038	0.90(0.84–0.97)	**0.007**
TRG				
0	1			
1	1.53(0.63–3.71)	0.352		
2	3.13(1.76–5.55)			
3	3.57(1.78–7.14)			
Smoke	1.67(1.06–2.63)	0.028	1.43(0.90–2.28)	0.127
Comorbidity	1.06(0.66–1.68)	0.82		

Abbreviation: NCRT, neoadjuvant chemoradiotherapy. E-LNS, examined lymph node station. ELN, examined lymph node. TRG, tumor regression grade. C-INDEX: 0.7578

### Effect of different LND strategies on DFS

As shown in Fig. 3C, D, patients with higher n-LDN (*P* = 0.042) or e-LNS (*P* = 0.0074) were significantly associated with better DFS in our patient population. The curve shows that there has been a marked increase in DFS with more lymph nodes removed. The univariable analysis and multivariable analysis are described in Supplementary Table 1. In univariable analysis, ypN stage and e-LNS were significantly associated with DFS. Based on the univariate analysis, the following variables were included in the model: differentiation, ypT, ypN, tumor length, e-LNS, and smoking. The results of the multivariable analysis showed that e-LNS (HR: 0.91; 95% CI, 0.84–0.98; *P* = 0.012) and ypN were independent predictors of better DFS.

### Recurrence patterns for different LND strategies

117 patients developed recurrences and metastasis during the follow-up, and the rates of loco-regional recurrence and distant metastasis were 16.5% (55/333) and 18.6% (62/333), respectively. The subgroup analysis showed that the high e-LNS group had a significantly lower loco-regional recurrence rate (13.3% vs. 23.1%, *P* = 0.036) than the low e-LNS group. There were a total of 21 cases of metastasis (19.4%) in the e-LNS≤ 9 group and 41 cases (18.2%) in the e-LNS ＞ 9 group, and no significant difference was found between the two groups. The details of the pattern of recurrent and metastatic tumors are shown in Table 3.

**Table 3:** Association Between Number of Lymph Node Station Dissections and Recurrence and Metastasis

Parameter	e-LNS ≤ 9	e-LNS > 9	*P* Value
	No. (%)	No. (%)	
Total	*N* = 108	*N* = 225	
LRR	25 (23.1)	30 (13.3)	0.036
Metastasis	21 (19.4)	41 (18.2)	0.906
LRR + Metastasis	11(10.2)	14(6.2)	0.266

Abbreviation: LRR, locoregional recurrence. e-LNS, examined lymph node station

### Postoperative complications

Postoperative complications included pulmonary infection, respiratory failure, unplanned reintubation, pleural effusion, pulmonary embolism, deep vein thrombosis, empyema, chylothorax, incision infection, anastomotic leakage, anastomotic stenosis, and injury of the recurrent nerve. The total incidence of postoperative complications was equal in different e-LNS groups (≤ 9 or > 9) (*P* = 0.363). The analysis for individual complications showed that the postoperative complication rate did not differ significantly between the low e-LNS group and the high e-LNS group, except for recurrent nerve injury (Supplementary Table 2).

## Discussion

The effect of LND on OS and DFS in patients treated with nCRT followed by esophagectomy was investigated in this study. The main finding revealed that a high number of resected e-LNS was linked to considerably improved OS and DFS compared to a low number of e-LNS. Furthermore, the e-LNS was an independent prognostic factor in our cohort, whereas the n-LND was not. The effects of nCRT on the number of collected LNs could be one reason for this finding. Previous studies suggested that nCRT may sterilize micrometastases because it removes fewer LNs at dissection than surgery alone for ESCC patients [12, 24]. Moreover, less recurrence was observed in the high e-LNS group. These results indicate that e-LNS could be used as a prognosticator for ESCC patients who received NCRT.

LNM is the most important prognostic factor for patients with ESCC, and various studies have confirmed that the number of LNMs is a relevant prognostic factor for OS and DFS in ESCC [25]. As a result, performing the correct assessment of the extent of LND not only leads to appropriate staging of ESCC but also leads to better survival outcomes. Three-field lymph node dissection is widely acknowledged as an appropriate procedure. While our study consistently included second-field lymph node dissection, the international community has not universally embraced third-field lymph node dissection as a standard procedure [26–28]. The lack of sufficiently sensitive investigations poses challenges in identifying patients suitable for third-field lymph node dissection [29]. Consequently, cervical lymph node dissection was selectively performed in cases with suspected cervical lymph node metastasis indicated by preoperative CT and ultrasound. Despite updates to the T category in the eighth edition of the AJCC Cancer Staging Manual, the N categorization remains unchanged from the seventh edition, relying on the number of metastatic LNs [10]. A minimum of 12 nodes should be removed during radical surgery for EC according to current guidelines [2, 10]. However, several studies have raised doubts about the reliability of the existing staging system. Based on the seventh AJCC TNM staging system, Yamasaki and Chen reported no significant difference between N2 and N3 survival probabilities [30–32]. The LN ratio (the ratio of metastatic to harvested LNs), station ratio (metastatic LN stations/e-LNSs), and number of negative LNs have been considered independent prognostic factors in various studies [3, 15, 20, 33, 34]. However, nodal staging systems based on ratios offer much opportunity for stage migration. Considering the substantial impact of nodal downstaging using nCRT, nodal dissection should be adjusted accordingly.

We observed no significant differences between the high and low n-LND groups, in contrast to the e-LNS group. This study showed that the number of LNDs recommended by the eighth edition of the AJCC Cancer Staging Manual did not reflect the OS of patients who underwent nCRT. We hypothesized that the correlation between the number of LNs and the number of LN stations after esophagectomy could be influenced by neoadjuvant therapy. A comparison study with 402 patients with EC (181 with adenocarcinoma and 221 with squamous cell carcinoma) revealed that neoadjuvant therapy reduces the frequency of LNMs and significantly modifies nodal localization and patterns [33]. Thus, we believe that e-LNS could serve as a better prognostic factor for patients with ESCC after receiving neoadjuvant therapy. Previous studies have elucidated the pivotal role of surgical quality in comparing survival outcomes among different surgical procedures [16]. In this study, we included patients from stage ypT0N0M0 to ypT4bN3M0. Our results show that over 50% of patients achieved successful LND of more than 9 lymph node stations (over 15 lymph nodes). Due to patient heterogeneity, surgeons should tailor LND strategies in clinical practice. This study is significant for introducing and emphasizing that actively pursuing comprehensive lymph node clearance during surgery may improve prognosis. Therefore, we believe that broadening the scope of LND in patients with ESCC following neoadjuvant therapy is crucial for improving patient prognosis.

Our study has some limitations. First, our study was a single-center retrospective study, wherein data pertaining to general demographic and disease-related information was extracted from the patients’ medical records at the hospital. This approach may have introduced selection bias, which is an inherent limitation of retrospective studies. Consequently, a comprehensive prospective study is imperative to substantiate the prognostic relevance of lymph node dissection (LND) for each lymph node (LN) station in individuals with ESCC who undergo neoadjuvant therapy followed by surgical intervention. Second, the pathological assessment of the removed LNs was dependent on the pathologists’ experience. However, all patients in this study underwent surgery at high-volume centers, thus avoiding potential bias resulting from different surgical skills and pathological experiences.

## Conclusion

This cohort study revealed an association between the extent of LND and overall survival, suggesting the therapeutic value of extended lymphadenectomy during esophagectomy. We found that enlarging the extent of LND is feasible and that patients with stages I-IVA ESCC who undergo nCRT followed by esophagectomy have a higher chance of long-term survival, and standard lymphadenectomy of at least 9 stations is strongly recommended. However, further studies are required to confirm these results.

### Electronic supplementary material

Below is the link to the electronic supplementary material.

Supplementary Material 1

## Data Availability

All datasets are available in the supplementary materials or upon request from the corresponding author for reasonable request.

## List of abbreviations

nCRT: Neoadjuvant chemoradiotherapy
LNS: Lymph node station
e-LNS: Examined lymph node station
ELN: Examined lymph node
TRG: Tumor regression grade
ESCC: Esophageal squamous cell carcinoma
LRR: Locoregional recurrence
OS: Overall survival
DFS: Disease-free survival
IQR: Interquartile range
CIs: Confidence intervals
HRs: Hazard ratios
LNM: Lymph node metastasis
LNDs: Lymph node dissections
n-LNDs: Number of lymph node dissections
e-LNSs: Station of lymph node dissections

## References

[1] Arnold M, Soerjomataram I, Ferlay J, Forman D (2015). Global incidence of oesophageal cancer by histological subtype in 2012. Gut.

[2] Shah MA, Kennedy EB, Catenacci DV, Deighton DC, Goodman KA, Malhotra NK, Willett C, Stiles B, Sharma P, Tang L (2020). Treatment of locally Advanced Esophageal Carcinoma: ASCO Guideline. J Clin Oncology: Official J Am Soc Clin Oncol.

[3] Kano K, Yamada T, Komori K, Watanabe H, Takahashi K, Fujikawa H, Numata M, Aoyama T, Tamagawa H, Yukawa N (2021). The Prognostic Value of Lymph Node ratio in locally Advanced Esophageal Cancer patients who received Neoadjuvant Chemotherapy. Ann Surg Oncol.

[4] Pennathur A, Gibson MK, Jobe BA, Luketich JD (2013). Oesophageal carcinoma. Lancet (London England).

[5] van der Schaaf M, Johar A, Wijnhoven B, Lagergren P, Lagergren J. Extent of lymph node removal during esophageal cancer surgery and survival. J Natl Cancer Inst 2015, 107(5). 10.1093/jnci/djv043 25748792

[6] Huang Y, Wang H, Luo G, Zhang Y, Wang L, Li K (2017). A systematic review and network meta-analysis of neoadjuvant therapy combined with surgery for patients with resectable esophageal squamous cell carcinoma. Int J Surg (London England).

[7] Leng XF, Daiko H, Han YT, Mao YS (2020). Optimal preoperative neoadjuvant therapy for resectable locally advanced esophageal squamous cell carcinoma. Ann N Y Acad Sci.

[8] Wong IYH, Lam KO, Zhang RQ, Chan WWL, Wong CLY, Chan FSY, Kwong DLW, Law SYK (2020). Neoadjuvant Chemoradiotherapy using cisplatin and 5-Fluorouracil (PF) Versus Carboplatin and Paclitaxel (CROSS Regimen) for esophageal squamous cell carcinoma (ESCC): a propensity score-matched study. Ann Surg.

[9] Bogoevski D, Onken F, Koenig A, Kaifi JT, Schurr P, Sauter G, Izbicki JR, Yekebas EF (2008). Is it time for a new TNM classification in esophageal carcinoma?. Ann Surg.

[10] Amin MB, Greene FL, Edge SB, Compton CC, Gershenwald JE, Brookland RK, Meyer L, Gress DM, Byrd DR, Winchester DP (2017). The Eighth Edition AJCC Cancer staging Manual: continuing to build a bridge from a population-based to a more personalized approach to cancer staging. Cancer J Clin.

[11] Rice TW, Ishwaran H, Ferguson MK, Blackstone EH, Goldstraw P (2017). Cancer of the Esophagus and Esophagogastric Junction: an Eighth Edition staging primer. J Thorac Oncology: Official Publication Int Association Study Lung Cancer.

[12] Koen Talsma A, Shapiro J, Looman CW, van Hagen P, Steyerberg EW, van der Gaast A, van Berge Henegouwen MI, Wijnhoven BP, van Lanschot JJ, Hulshof MC (2014). Lymph node retrieval during esophagectomy with and without neoadjuvant chemoradiotherapy: prognostic and therapeutic impact on survival. Ann Surg.

[13] Miyata H, Sugimura K, Yamasaki M, Makino T, Tanaka K, Morii E, Omori T, Yamamoto K, Yanagimoto Y, Yano M (2019). Clinical impact of the location of Lymph Node Metastases after Neoadjuvant Chemotherapy for Middle and Lower thoracic esophageal Cancer. Ann Surg Oncol.

[14] Robb WB, Dahan L, Mornex F, Maillard E, Thomas PA, Meunier B, Boige V, Pezet D, Le Brun-Ly V, Bosset JF (2015). Impact of neoadjuvant chemoradiation on lymph node status in esophageal cancer: post hoc analysis of a randomized controlled trial. Ann Surg.

[15] Yuan Y, Hong HG, Zeng X, Xu LY, Yang YS, Shang QX, Yang H, Li Y, Li Y, Wu ZY (2019). Lymph Node Station-based nodal staging system for esophageal squamous cell carcinoma: a large-scale Multicenter Study. Ann Surg Oncol.

[16] Liu F, Yang W, He Y, Yang W, Chen L, Xu R, Liu Z, Ke J, Hou B, Zhang L et al. Surgical quality determines the long-term survival superiority of right over left thoracic esophagectomy for localized esophageal squamous cell carcinoma patients: a real-world multicenter study. Int J Surg 2023. 10.1097/JS9.0000000000000897 PMC10871567 37983771

[17] Wang D, Smit JK, Zwaan E, Muijs CT, Groen H, Hollema H, Plukker JT (2013). Neoadjuvant therapy reduces the incidence of nodal micrometastases in esophageal adenocarcinoma. Am J Surg.

[18] Altorki NK, Zhou XK, Stiles B, Port JL, Paul S, Lee PC, Mazumdar M (2008). Total number of resected lymph nodes predicts survival in esophageal cancer. Ann Surg.

[19] Mulligan ED, Dunne B, Griffin M, Keeling N, Reynolds JV (2004). Margin involvement and outcome in oesophageal carcinoma: a 10-year experience in a specialist unit. Eur J Surg Oncology: J Eur Soc Surg Oncol Br Association Surg Oncol.

[20] Peng J, Wang WP, Dong T, Cai J, Ni PZ, Chen LQ (2016). Refining the nodal staging for esophageal squamous cell Carcinoma based on Lymph Node stations. Ann Thorac Surg.

[21] Pultrum BB, Honing J, Smit JK, van Dullemen HM, van Dam GM, Groen H, Hollema H, Plukker JT (2010). A critical appraisal of circumferential resection margins in esophageal carcinoma. Ann Surg Oncol.

[22] Zhu Z, Chen H, Yu W, Fu X, Xiang J, Li H, Zhang Y, Sun M, Wei Q, Zhao W (2014). Number of negative lymph nodes is associated with survival in thoracic esophageal squamous cell carcinoma patients undergoing three-field lymphadenectomy. Ann Surg Oncol.

[23] Liang W, He J, Shen Y, Shen J, He Q, Zhang J, Jiang G, Wang Q, Liu L, Gao S (2017). Impact of examined Lymph Node count on Precise Staging and Long-Term Survival of Resected Non-small-cell Lung Cancer: a Population Study of the US SEER database and a Chinese multi-institutional Registry. J Clin Oncology: Official J Am Soc Clin Oncol.

[24] Matsuda S, Kawakubo H, Takeuchi H, Mayanagi S, Irino T, Fukuda K, Nakamura R, Wada N, Kitagawa Y (2021). Prognostic impact of thoracic duct lymph node metastasis in esophageal squamous cell carcinoma. Annals of Gastroenterological Surgery.

[25] Yamasaki M, Miyata H, Miyazaki Y, Takahashi T, Kurokawa Y, Nakajima K, Takiguchi S, Mori M, Doki Y (2014). Evaluation of the nodal status in the 7th edition of the UICC-TNM classification for esophageal squamous cell carcinoma: proposed modifications for improved survival stratification: impact of lymph node metastases on overall survival after esophagectomy. Ann Surg Oncol.

[26] Ji X, Cai J, Chen Y, Chen LQ (2016). Lymphatic spreading and lymphadenectomy for esophageal carcinoma. World J Gastrointest Surg.

[27] Li H, Zhang ZR (2019). Current status and future direction of lymph node dissection in radical surgery for esophageal cancer. J Thorac Dis.

[28] Li K, Du K, Liu K, Nie X, Li C, He W, Li K, Wang C, Li Z, Zheng K et al. Impact of two–field or three–field lymphadenectomy on overall survival in middle and lower thoracic esophageal squamous cell carcinoma: a single–center retrospective analysis. Oncol Lett 2023, 25(5). 10.3892/ol.2023.13774 PMC10091183 37065785

[29] Udagawa H (2020). Past, present, and future of three-field lymphadenectomy for thoracic esophageal cancer. Annals of Gastroenterological Surgery.

[30] Chen SB, Weng HR, Wang G, Yang JS, Yang WP, Liu DT, Chen YP, Zhang H (2013). Prognostic factors and outcome for patients with esophageal squamous cell carcinoma underwent surgical resection alone: evaluation of the seventh edition of the American Joint Committee on Cancer staging system for esophageal squamous cell carcinoma. J Thorac Oncology: Official Publication Int Association Study Lung Cancer.

[31] Guo X, Wang Z, Yang H, Mao T, Chen Y, Zhu C, Yu Z, Han Y, Mao W, Xiang J (2023). Impact of Lymph Node Dissection on Survival after Neoadjuvant Chemoradiotherapy for locally advanced esophageal squamous cell carcinoma: from the results of NEOCRTEC5010, a Randomized Multicenter Study. Ann Surg.

[32] Tan Z, Ma G, Yang H, Zhang L, Rong T, Lin P (2014). Can lymph node ratio replace pn categories in the tumor-node-metastasis classification system for esophageal cancer?. J Thorac Oncology: Official Publication Int Association Study Lung Cancer.

[33] Castoro C, Scarpa M, Cagol M, Ruol A, Cavallin F, Alfieri R, Zanchettin G, Rugge M, Ancona E (2011). Nodal metastasis from locally advanced esophageal cancer: how neoadjuvant therapy modifies their frequency and distribution. Ann Surg Oncol.

[34] Shao Y, Geng Y, Gu W, Ning Z, Huang J, Pei H, Jiang J (2016). Assessment of Lymph Node ratio to replace the pN categories system of classification of the TNM System in Esophageal squamous cell carcinoma. J Thorac Oncology: Official Publication Int Association Study Lung Cancer.

