# Supplementary material for: Increased resected lymph node stations improved survival of esophageal squamous cell carcinoma
Source: BMC Cancer. 2024 Feb 5;24:177. doi: 10.1186/s12885-024-11886-7 (PMC10845591; doi:10.1186/s12885-024-11886-7)
Supplement: Supplementary file 1 — Supplementary Material 1 [file 12885_2024_11886_MOESM1_ESM.docx]

Supplementary table 1. Univariate and multivariate Cox regression analysis of prognosis for DFS in nCRT patients

|  |  | Univariable Analysis |  |  | Multivariable Analysis |  |
| --- | --- | --- | --- | --- | --- | --- |
| Prognostic Factor |  | HR (95% CI) | P Value |  | HR (95% CI) | P Value |
| Age, years |  | 0.98(0.95-0.99) | 0.091 |  |  |  |
| Sex |  | 1.61(0.87-2.97) | 0.128 |  |  |  |
| Differentiation |  |  |  |  |  |  |
| Gx |  | 1 |  |  | 1 |  |
| G1 |  | 1.29(0.38-4.40) | 0.681 |  | 0.42(0.06-2.74) | 0.362 |
| G2 |  | 2.37(1.29-4.33) | 0.005 |  | 0.45(0.09-2.33) | 0.342 |
| G3 |  | 3.85(2.17-6.70) | 0.000 |  | 0.64(0.12-3.32) | 0.594 |
| ypT |  |  |  |  |  |  |
| 0 |  | 1 |  |  | 1 |  |
| 1 |  | 1.49(0.61-3.61) | 0.382 |  | 0.56(0.08-3.9) | 0.557 |
| 2 |  | 1.99(0.92-4.29) | 0.077 |  | 0.65(0.08-5.06) | 0.677 |
| 3 |  | 4.10(2.33-7.21) | 0 |  | 1.09(0.15-7.64) | 0.934 |
| ypN |  |  |  |  |  |  |
| 0 |  | 1 |  |  | 1 |  |
| 1 |  | 3.33(2.01-5.52) | 0 |  | 3.16(1.84-5.4) | **0** |
| 2 |  | 3.62(1.92-6.83) | 0 |  | 3.40(1.67-6.94) | **0.001** |
| 3 |  | 17.69(7.83-39.96) | 0 |  | 14.29(5.29-38.64) | **0** |
| Pathological Stage |  |  |  |  |  |  |
| 0 |  | 1 |  |  |  |  |
| 1 |  | 2.61(1.27-5.38) | 0.009 |  |  |  |
| 2 |  | 4.74(2.72-8.29) | 0 |  |  |  |
| 3 |  | 23.24(9.36-57.68) | 0 |  |  |  |
| Tumor location |  |  |  |  |  |  |
| Proximal third |  | 1 |  |  |  |  |
| Middle third |  | 0.92(0.44-1.93) | 0.819 |  |  |  |
| Distal third |  | 1.04(0.65-1.66) | 0.868 |  |  |  |
| Tumor length, cm |  | 1.28(1.18-1.39) | 0 |  | 1.21 (1.05-4.10) | 0.009 |
| ELN  E-LNS |  | 0.99(0.96-1.01)  0.93(0.87-0.99) | 0.361 0.034 |  | 0.91(0.84-0.98) | **0.012** |
| TRG |  |  |  |  |  |  |
| 0 |  | 1 |  |  |  |  |
| 1 |  | 1.53(0.63-3.71) | 0.347 |  |  |  |
| 2 |  | 3.13(1.76-5.55) | 0 |  |  |  |
| 3 |  | 3.57(1.78-7.14) | 0 |  |  |  |
| Smoke |  | 1.74(1.1-2.75) | 0.017 |  | 1.36(0.85-2.17) | 0.203 |
| Comorbidity |  | 1.09(0.69-1.73) | 0.715 |  |  |  |

Abbreviation: NCRT, neoadjuvant chemoradiotherapy. E-LNS, examined lymph node station. ELN, examined lymph node. TRG, tumor regression grade. C-INDEX: 0.7557.

Supplementary Table 2. Association Between resected Lymph Node Station and Postoperative Complications in the nCRT Patients

|  | ELNS≤9(N=108) | ELNS>9(N=225) |  |
| --- | --- | --- | --- |
| Characteristics | No. (%) | No. (%) | P Value |
| Total | 34(34.5) | 72(32.0) | 1.00 |
| Pulmonary infection | 26(24.1) | 51(22.7) | 0.783 |
| Respiratory failure | 0 | 3(1.3) | 0.554 |
| Unplanned re-intubation | 6(5.6) | 14(6.2) | 1 |
| Pleural effusion | 14(13.0) | 32(14.2) | 0.866 |
| pulmonary embolism | 0 | 1(0.4) | 1 |
| Deep vein thrombosis | 0 | 1(0.4) | 1 |
| empyema | 0 | 5(2.2) | 0.179 |
| Chylothorax | 0 | 4(1.8) | 0.309 |
| Incision infection | 8(7.4) | 12(5.3) | 0.467 |
| Anastomotic leakage | 7(6.5) | 11(4.9) | 0.607 |
| Anastomotic stenosis | 3(2.8) | 3(1.3) | 0.394 |
| Injury of recurrent nerve | 1(0.9) | 14(6.2) | 0.044 |
